# Non-marginal jejunal ulcer perforation following Roux-en-Y gastric bypass

**DOI:** 10.1093/jscr/rjac112

**Published:** 2022-03-26

**Authors:** Luke D Fairweather, Toan D Pham

**Affiliations:** Department of General Surgery, Western Health, Melbourne, Australia; Department of General Surgery, Western Health, Melbourne, Australia

**Keywords:** bariatric surgery, gastric bypass, general surgery, jejunal perforation, Roux-en-Y

## Abstract

We present a rare case of a jejunal ulcer perforation in the alimentary limb ~15 cm distal to the gastro-jejunal anastomosis on the background of a previous Roux-en-Y gastric bypass (RYGB) 4 months prior to presentation. Marginal ulcer is the most common cause of jejunal perforation following RYGB. However, this is usually confined to the first few centimetres, and the incidence is highest within the first month following surgery. Other risk factors include smoking and non-steroidal anti-inflammatory drug use, *Helicobacter pylori* infection, trauma, foreign body ingestion, Crohn’s disease, typhoid, tuberculosis and malignancy. This case does not possess any of these risk factors and thus represents a unique presentation. Not all jejunal ulcers will present with classical risks factors but still will need to be excluded, given their life-threatening nature. Also, the whole alimentary limb can be susceptible to ulceration; therefore, a thorough investigation of this limb is important to exclude perforation.

## INTRODUCTION

Peptic ulcer perforation, while common in the stomach or duodenum, is rarely seen in the jejunum. Most non-*Helicobacter pylori* ulcers are either associated with alcohol intake or non-steroidal anti-inflammatory drug (NSAID) use. In the absence of these risk factors, perforation is rare. Other risk factors for gastrointestinal perforation include instrumentation, trauma, ingested foreign body, Crohn’s disease, diverticular disease, smoking, typhoid, tuberculosis and malignancy [1–3]. Marginal ulcers are the most common cause of jejunal perforation following Roux-en-Y gastric bypass (RYGB) and are reported up to 2% [4]. However, marginal ulcers usually confined to the first few centimetres after an anastomosis [5], and the incidence is highest within the first month following surgery. The exact aetiology is uncertain, however, is likely multifactorial with the ischemia and acid hypersecretion being possible causative factors [2]. We present a rare case of a non-marginal ulcer perforation of the alimentary limb following a RYGB.

## CASE REPORT

A 39-year-old female presented with sudden-onset severe left upper quadrant abdominal pain radiating to left shoulder tip and recumbent shortness of breath. The patient had undergone an uncomplicated RYGB for weight loss 4 months prior. Other relevant history includes smoking cessation 6 months prior and no recent NSAID or alcohol intake. She took no regular medications. The patient was tachycardic with a heart rate of 150 beats per minute and tachypnoeic at 35 breaths per minute, with upper abdominal peritonism on examination.

Investigations revealed a normal haemoglobin of 142 g/l and normal white cell count of 7.4 × 10^9^/l. C-reactive protein was mildly elevated at 22 mg/l. Lipase was normal at 17 units/l (reference range: 0–60). Bicarbonate was low at 19 mmol/l, however, the remaining renal function and liver function tests were normal. A venous blood gas demonstrated a metabolic acidosis with a compensatory respiratory alkalosis with a pH of 7.39 and a base deficit of 8.9 mmol/l. Lactate was elevated at 5 mmol/l. Other blood investigations showed a normal international normalized ratio and a negative serum Beta-HCG.

**Figure 1:**
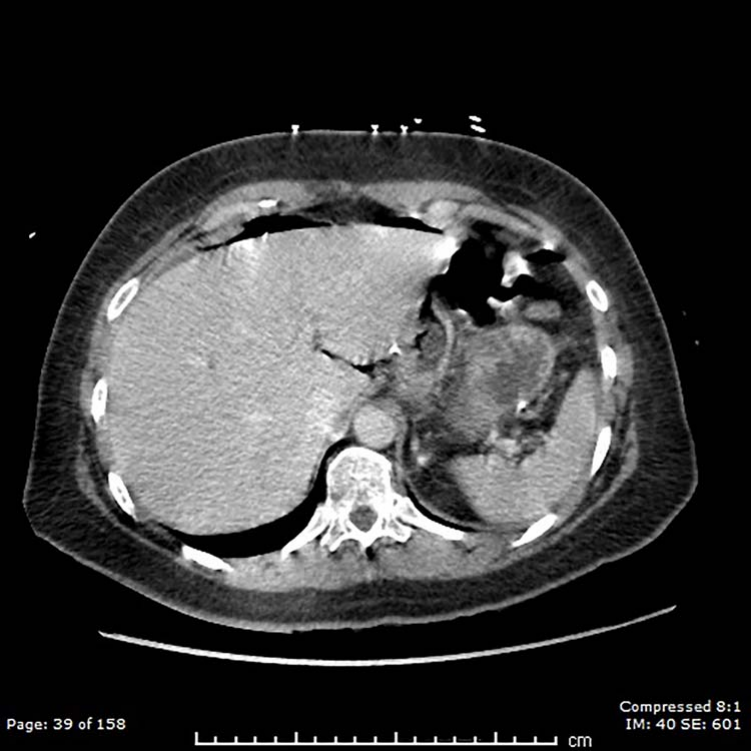
Free gas on CT.

**Figure 2:**
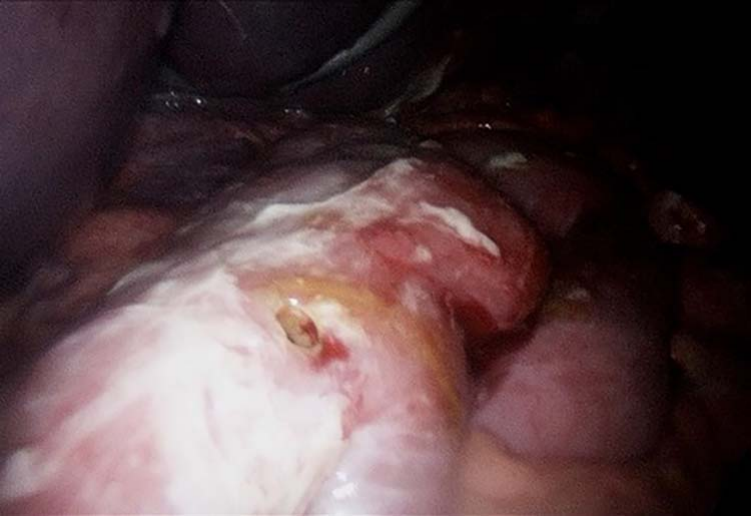
Alimentary limb perforation at laparoscopy.

A computed tomography (CT) scan of the abdomen and pelvis showed moderate volume free gas without a clear source (Fig. 1). At diagnostic laparoscopy, there was four-quadrant purulent peritonitis and a 5-mm anterior perforation in the alimentary limb located ~15 cm from the gastrojejunostomy (Fig. 2). This was repaired laparoscopically with primary closure after excision of the ulcer edges; the histology revealed no malignancy nor *Helicobacter pylori*. The patient had an uneventful recovery and was discharged on post-operative day 5.

## DISCUSSION

Our case is unique as it does not possess any of the above risk factors and is also at an unusual location at 15 cm distal to the gastro-jejunal anastomosis. This case illustrates several important discussion points. In early phases, upper gastrointestinal perforation can present counterintuitively with patient movement as opposed to the lack of movement expected to develop in the coming hours with the onset of generalized peritonism. Not all jejunal ulcers will present with classical risks factors but will still need to be excluded, given their life-threatening nature. Also, the whole alimentary limb can be susceptible to ulceration; therefore, a thorough investigation of this limb is important to exclude perforation.

Free gas on imaging indicates viscus perforation, and if associated with peritonism, mandates surgical exploration, particularly if the location of perforation remains unclear. For patients with acute perforation and peritonism, emergency surgery and closure is the standard of care, however, this becomes more complex in non-acute settings. Rapid diagnosis is essential to facilitate good patient outcomes and it is important not to delay treatment for further investigation. Resuscitation can be continued in the operation theatre. Early surgical management minimizes contamination and decreases the associated risks of sepsis. Small perforations may be oversewed or repaired with an omental or falciform patch, whereas larger perforations may require a wedge or segmental resection. In experienced hands, laparoscopic management allows a faster post-operative recovery [6].

Several factors have likely contributed to a good outcome for our patient. These include young age, absence of significant co-morbidities and early sepsis control.

## CONFLICT OF INTEREST STATEMENT

None declared.

## FUNDING

None.
